# Aboard the Good Ship Verona

**DOI:** 10.4269/ajtmh.18-0709

**Published:** 2018-10-08

**Authors:** Claire Panosian Dunavan

**Affiliations:** Division of Infectious Diseases; University of California; Los Angeles, CA 90095-1688; E-mail: cpanosian@mednet.ucla.edu

“A youth with a heartbeat cannot circle most of the globe for a year without being profoundly altered,” writes tropical medicine colleague Barnett Cline in the preface of “*The Wind Blew Me There—Memories of a Ship’s Surgeon Aboard the Barquentine Verona*.” Were truer words ever uttered?

In 1965, Cline had not yet chosen global public health as his calling when boarding the 138-foot schooner as a sole medic and fellow tar for a crew of twenty-five. At the end of his voyage, the world looked a lot different; so did his future career. Cline would subsequently complete a PhD in epidemiology at UC Berkeley; work on dengue and schistosomiasis for CDC’s field lab in San Juan, Puerto Rico; expand his work (mainly on helminthiases) to sites as far-flung as Egypt, Cameroon, and Malaysia; and eventually serve as Professor and Chair of Tropical Medicine at Tulane University’s School of Public Health and Tropical Medicine. In short, his life-adventures were just beginning when his marine odyssey ended in an ancient Greek village where he and his wife Nancy later owned a home for thirty-five years.

In one respect, however, ascending Verona’s gangway seems a laudable act of courage when viewed through the lens of time. Four years earlier, the ship’s skipper had survived a terrifying white squall, which not only claimed his square-rigged brig “Albatross,” but also the lives of his physician-wife Alice and four high-school-aged sailors. Although Cline immediately took to Christopher Sheldon when they first met in Bogota (at the time, Cline was working in Bolivia as a Peace Corps doctor and Sheldon was the Peace Corps director for Colombia), as independent accounts would later attest, the skipper had a darker side.

After sharing this eye-popping intel, Cline—a former ASTMH President—interweaves modern reunions with Verona shipmates with captivating stories of people and cultures he encountered on a long and exotic itinerary. His ports of call included the Galapagos, Pitcairn Islands, French Polynesia, Cook Islands, Samoa, Fiji, New Hebrides, Papua New Guinea, Taiwan, Hong Kong, Thailand, Malaysia, Andaman Islands, India, Sri Lanka, Maldives, Yemen, Saudi Arabia, Egypt, Lebanon, Greece, Malta, Spain, and Portugal. That the book’s chronology is not precisely linear adds to its freshness.

Then there’s the pure adventure and thrill of this travelogue-cum-memoir, nicely “teased” in an Amazon blurb.

“…fifty years later, Cline recalls performing emergency surgery while on the Galapagos Islands, dodging the arrows of hostile natives in the Bay of Bengal, experiencing a mutiny mid-Pacific, and acquiring a cave house overlooking the Aegean Sea on Santorini, Greece. Cline also shares medical moments that tested his education and gave him invaluable real-world experience in emergency and tropical medicine.”

Since further details might spoil the delight of reading “*The Wind Blew Me There*,” let me simply say: this is a one-of-a-kind memoir well worth enjoying and sharing! The following interview with its author was conducted in July 2018.

## Let’s start with more medical bio and how this remarkable trip informed your later choice of career.

At my medical school—Baylor—the reigning gods were DeBakey and Cooley. If you weren’t transplanting hearts, you weren’t a real doctor. So I was initially caught up in the excitement of surgery, and I held on to that interest through my rotating internship at Tulane. At that point, I was accepted into Tulane’s surgery program. I was supposed to complete two years in the Peace Corps to fulfill my military requirement—remember I couldn’t join the Navy because of a high-frequency hearing loss I didn’t know I had until my pre-entry physical examination—and then do the residency.

But now I’ve got to step back because tropical medicine seeds were actually planted in Panama as a senior medical student. Back then I had a two-month fellowship at the Gorgas Memorial laboratory, which included a lot of field exposure and some clinical work and also introduced me to Karl Johnson and Ron McKenzie, who were both members of the Society. My Peace Corps experience in Bolivia reinforced my interest in working in remote areas, and the year at sea gave me added time to grow and reflect. Eventually, I decided that practicing surgery would not be consistent with the big-picture career I wanted.

Here’s a final connection with the Society. It was Karl Johnson [another former ASTMH President and founding Director of CDC’s Special Pathogens Unit] who saw me off as we sailed into the Pacific. I think he would have liked to have climbed aboard as well! Later, in 1993, when I was greatly honored to serve as ASTMH President, Karl roasted me before my Presidential address. That’s when he described me as a risk-taker and showed a photograph of my boarding the Verona. Before that moment, I had never thought of myself as a risk-taker.

## Please share the journey’s most significant revelation and its scariest moment.

Well, I don’t think there was a single, over-arching revelation that came to me during the year. But every port of call exposed me to a new culture, language, cuisine, geography, and history. It was just fascinating to absorb all of that. In addition, there was the sailing experience itself. When I was on night-watch from midnight to four, or from four to eight in the morning, I felt connected to mariners through the ages. I’d see whales and porpoises frolicking—water spouts and active volcanos—I also did a fair bit of free-diving and scuba-diving. It was a never-ending set of experiences.

Some 24-hour gale-force winds in the South China Sea were our biggest scare. Of course, at the time, we were really too busy to be scared; it was only after the winds subsided and we thought back that we were terrified. Waves were crashing all over the place and in order to be at the wheel or on deck for any purpose, you had to harness yourself so you wouldn’t be washed overboard.

## I can’t help asking about the skipper. Given the *Albatross’s* history, did you have private trepidations about him?

At the beginning, I was naive about the skipper, and I had full confidence in him. And maybe I didn’t want to see the flaws that later became pretty obvious. It took a while for the whole crew, me included, to realize we were at risk. Finally, when we were approaching one of the French Polynesian islands and he neglected to give the order to drop sail because he was totally inebriated, we almost crashed into the shore. So that’s what finally woke us up. We couldn’t ignore that.

[Addendum: After this event, with the whole crew present, the ship’s engineer confronted Sheldon, explaining that the entire company would exit in Hong Kong if Sheldon did not refrain from all further drinking. He did.]

## Please describe the process of writing your memoir. What advice can you share with colleagues who might someday follow your lead?

It was a five-year process. First came my partial retirement, and then complete retirement, which gave me the time not just to reflect but to look at the thousands of photographs I shot on the trip. After digitizing three hundred, I could actually sit down and work with them in my computer, which was a critical step. But discovering my journal was even more important. After I found it and started reading, it was like I had gone to the moon! Finally, I found letters my parents had kept in a box. All this plus my detailed photographic notes were the material I needed.

But the real catalysts were the photographs, because each one brought back so many memories. One in particular of a crewmate made me wonder: where is that guy? So I actually found him living in the mountains of western Honduras and arranged a trip to meet with him. That experience, in particular, convinced me that my book could go beyond high adventure and present special individuals and characters.

A final bit of advice? Keep careful notes! If you don’t, memories can be treacherous.

## Now that several months have passed since its publication, what reactions to “*The Wind Blew Me There*” have surprised or pleased you the most?

The most satisfying responses I’ve gotten are from strangers. For example, one gentleman told me he enjoyed the book so much that he gave a copy to his nephew, hoping it would encourage him to take a year, get off the merry-go-round, and expand his world.

## That’s a nice segue to a final comment from a long-time medical friend who circumnavigated the globe in the 1980s. Here’s what he wrote after reading your book:

“The fact that the story took place in the mid-60s, long before social media, Instagram, Snapchat, Wi-Fi, and the ‘see-me-now’ world adds to its special allure. It harks back to a simpler, more lovely world where adventure was more genuine and, perhaps, more plentiful.

Then there was only the sextant and dead reckoning for navigation versus the constant, exact whereabouts of anyone, anytime, anywhere on earth.”

## What’s your reaction?

I absolutely agree. After I reflected on Bob Lux’s words, I realized it’s absolutely true that the voyage I took—for the exact reasons he stated—could not be replicated today. And that makes me appreciate all the more the uniqueness of my experience.

